# Therapeutic Trem2 activation ameliorates amyloid-beta deposition and improves cognition in the 5XFAD model of amyloid deposition

**DOI:** 10.1186/s12974-020-01915-0

**Published:** 2020-08-14

**Authors:** Brittani R. Price, Tiffany L. Sudduth, Erica M. Weekman, Sherika Johnson, Danielle Hawthorne, Abigail Woolums, Donna M. Wilcock

**Affiliations:** 1 Sanders-Brown Center on Aging, College of Medicine, University of Kentucky, 800 S Limestone St, Lexington, KY 40536 USA; 2 Department of Physiology, University of Kentucky, Lexington, KY 40536 USA

**Keywords:** TREM2, Trem2, Alzheimer’s disease, Beta-amyloid, Immunotherapy, Neuroinflammation, Immuno-neurology

## Abstract

**Background:**

Triggering receptor expressed on myeloid cell-2 (TREM2) is a lipid and lipoprotein binding receptor expressed by cells of myeloid origin. Homozygous TREM2 mutations cause early onset progressive presenile dementia while heterozygous, point mutations triple the risk of Alzheimer’s disease (AD). Although human genetic findings support the notion that loss of TREM2 function exacerbates neurodegeneration, it is not clear whether activation of TREM2 in a disease state would result in therapeutic benefits. To determine the viability of TREM2 activation as a therapeutic strategy, we sought to characterize an agonistic Trem2 antibody (AL002a) and test its efficacy and mechanism of action in an aggressive mouse model of amyloid deposition.

**Methods:**

To determine whether agonism of Trem2 results in therapeutic benefits, we designed both intracranial and systemic administration studies. 5XFAD mice in the intracranial administration study were assigned to one of two injection groups: AL002a, a Trem2-agonizing antibody, or MOPC, an isotype-matched control antibody. Mice were then subject to a single bilateral intracranial injection into the frontal cortex and hippocampus and euthanized 72 h later. The tissue from the left hemisphere was histologically examined for amyloid-beta and microglia activation, whereas the tissue from the right hemisphere was used for biochemical analyses. Similarly, mice in the systemic administration study were randomized to one of the aforementioned injection groups and the assigned antibody was administered intraperitoneally once a week for 14 weeks. Mice underwent behavioral assessment between the 12- and 14-week timepoints and were euthanized 24 h after their final injection. The tissue from the left hemisphere was used for histological analyses whereas the tissue from the right hemisphere was used for biochemical analyses.

**Results:**

Here, we show that chronic activation of Trem2, in the 5XFAD mouse model of amyloid deposition, leads to reversal of the amyloid-associated gene expression signature, recruitment of microglia to plaques, decreased amyloid deposition, and improvement in spatial learning and novel object recognition memory.

**Conclusions:**

These findings indicate that Trem2 activators may be effective for the treatment of AD and possibly other neurodegenerative disorders.

## Background

Alzheimer’s disease (AD) and related dementias have no disease-modifying treatments at this time and represent a looming public health crisis given the continually growing aging population [1]. To date, targeting the beta-amyloid (Aβ) peptide within amyloid plaques remains a leading candidate for therapeutic targeting; however, efforts have been hampered by lack of efficacy and/or significant adverse events [2–4]. As our understanding of the biological processes involved in AD continues to evolve, neuroinflammatory processes have gained significant interest and are currently at the forefront of research. While inflammation has long been understood to be a part of the brain response to plaque and tangle pathology associated with AD, the recent identification of mutations in the triggering receptor expressed on myeloid cell-2 (TREM2), a single transmembrane receptor expressed on the surface of microglia, has provoked further excitement surrounding immune modulation as a candidate therapeutic target for AD [5, 6].

TREM2 binds lipids, glycolipids, lipoproteins, and apolipoproteins including ApoE, clusterin/ApoJ, and, importantly, Aβ [7–10]. Ligand binding results in TREM2 co-clustering with the immunoreceptor tyrosine-based activation motif (ITAM) containing transmembrane protein TYROBP/DAP12 and subsequent phosphorylation of tyrosine residues of DAP12 by Src family kinases. Phosphorylated DAP12 in turn recruits spleen tyrosine kinase (Syk) leading to activation of downstream signaling events, including intracellular Ca^2+^, flux [11], activation of extracellular signal-regulated kinases (ERK) and of phosphoinositide 3-kinase (PI3K) [12], and nuclear translocation of the transcription factor nuclear factor of activated T cells (NFAT) [7]. The resulting changes in gene expression alongside post-transcriptional modifications induce an increased cellular response to colony-stimulating factor [13], actin reorganization [11], process extension [14], cytokine release [15, 16]**,** survival [17, 18], proliferation [13], migration [19], and phagocytosis [20] in Trem2 expressing dendritic cells (DC) [16], tissue macrophages [21], osteoclasts [22], and microglia [23].

Individuals homozygous for loss of function mutations in TREM2 [24] invariably develop polycystic lipomembranous osteodysplasia with sclerosing leukoencephalopathy (PLOSL), also known as Nasu-Hakola disease (NHD), which manifests as early-onset presenile dementia with frequent bone cysts [25], or as frontotemporal dementia (FTD) [26] with seizures and corpus callosum atrophy. Heterozygous TREM2 point mutations, which reduce either ligand binding [9] or cell surface expression [27], are associated with a number of pathologies including an overall reduction in the number of microglia associated with amyloid plaques as well as an inability of the remaining microglia to compact beta-amyloid deposits and form a barrier between Aβ plaques and neurons [14]. Additionally, heterozygous TREM2 point mutations have been associated with an increase in the number of phospho-tau positive, dystrophic neurites [28], and increased tau in cerebrospinal fluid [29]. Notably, these mutations also double the rate of brain atrophy [28, 30], triple the risk of AD [5, 6], and decrease the age of AD onset by 3–6 years [31].

Similarly, Trem2 homozygous (Trem2-/-) or heterozygous (Trem2+/-) deficient wildtype (WT) or AD mice also display multiple microglia pathologies, including reduced numbers of microglia, increased apoptotic cell death, nonreactive microglia cell morphology, the inability to coalesce around and compact beta amyloid plaques [32], abnormal AD gene expression signature [33], age-dependent inability to reduce total Aβ plaque load [34], and an inability to support Aβ antibody-mediated beta amyloid plaque clearance [35]. Furthermore, *Trem2 (-/-) microglia fail to fully activate into phagocytic,* disease-associated microglia and to express the associated gene signature *in AD mice* [36]. Although human genetics indicate that loss of TREM2 function is detrimental, there is no evidence that TREM2 gain of function would be beneficial. TREM2 pathology, like Aβ pathology [37], may begin decades before clinical symptoms arise, rendering intervention in patients diagnosed with AD ineffective. Likewise, the activation of TREM2 may result in indiscriminate and harmful stimulation of microglia and other innate immune cells. To determine the viability of TREM2 activation as a therapeutic strategy, we sought to identify and characterize an agonistic TREM2 antibody and test its efficacy and mechanism of action in an aggressive mouse model of amyloid deposition.

## Methods

### Animals

Male 5XFAD transgenic mice overexpressing the K670N/M671L (Swedish), I716V (Florida), and V717I (London) mutations in human APP (695), as well as M146L and L286V mutations in human PS1 [38] were aged to 3.5 months at Taconic and transferred to University of Kentucky. The study was approved by the University of Kentucky Institutional Animal Care and Use Committee and conformed to the National Institutes of Health Guide for the Care and Use of Animals in Research. All studies were performed blinded. Alector provided the antibodies coded. The mice were also coded and randomized into each group. Only upon completion of the data analysis were the groups unblinded. Mice were genotyped for the retinal degeneration (rd) mutation post-mortem. We found that there were six total mice in the systemic study that were homozygous for the rd mutation. Five of these were wildtype mice, and one was a 5XFAD mouse in the AL002a group. These mice were excluded from the study and are not represented in the sample sizes below.

### Antibodies for treatment

TREM2tm1(KOMP) Vlcg mice were immunized by ImmunoPrecise with hTREM2-Fc recombinant protein using standard procedures. Bleed titers were evaluated in in vitro assays, such as ELISA or FACS. Animals with a good immune response to the antigen were selected for fusion and given a final i.v. boost of antigen without adjuvant. Lymphocytes were isolated from the immunized animals and fused with mouse myeloma cells using polyethylene glycol (PEG 1500; Roche, 10783641001) according to the manufacturer’s instructions. Fused cells were plated into semisolid methylcellulose-based medium containing hypoxanthine, aminopterin, and thymidine for 10–12 days, allowing for single-step cloning and hybridoma selection. Single colonies were picked and transferred to 96-well plates containing culture medium with hypoxanthine-thymidine and grown for 4–5 days until mid-log-phase growth was reached. Supernatants were screened by ELISA for IgG production, isotype, and antigen specificity and by FACS for binding to a native antigen on cells. Positive hybridoma clones were subcloned using a single-step cloning system to ensure monoclonality, and the subclone supernatants were rescreened by FACS to confirm specificity. Final subclones were expanded in culture, and the supernatants were purified by Protein-A affinity chromatography. Purified antibodies were tested in vitro for specificity, functional activity, binding affinity, and epitope binning. The AL002a antibody does not bind to human TREM2, only murine. Anti-MOPC was obtained commercially and is also an IgG1 isotype (BioXCell, Lebanon, NH).

### Murine macrophages

Murine bone marrow precursor cells from Trem2-KO and Trem2-WT (Alector colony) were obtained by flushing tibial and femoral marrow cells with cold PBS containing 2% fetal bovine serum (FBS). Red blood cells were lysed using ammonium—chloride—potassium (ACK) lysing buffer, washed twice with 2% FBS/PBS, and re-suspended in complete media (RPMI, 10% FBS, Pen/Strep, l-glutamine, non-essential amino acid) with 50 ng/mL murine M-CSF (m-MCSF) to differentiate macrophages for 6 days.

For FACS analysis of AL002a binding to BMMs, cells were washed in FACS buffer (PBS + 2% FBS) and incubated with either AL002a, rat anti-Trem2 (R&D Systems) as a positive control, or murine IgG1 isotype (BD Biosciences) as a negative control in FACS buffer for 1 h on ice. Cells were washed three times in FACS buffer and spun. Goat anti-mouse APC conjugated secondary antibody was added in FACS buffer (BD Biosciences, 1:100), and cells were incubated on ice for 30 min. Cells were again washed as before, re-suspended in FACS buffer, and analyzed on a BD Canto Flow Cytometer.

### Biochemistry

Before stimulation, BMM were incubated for 4 h in 1% serum RPMI. For Syk immunoprecipitation 5 × 10^6^ cells were used, and for Trem2 immunoprecipitation 15 × 10^6^ cells were used. Cells were then incubated on ice for 15 min with 1 μg of AL002a or MOPC per 1 x 10^6^ cells. Cells were then washed and lysed with lysis buffer (1% v/v NP-40%, 50 Mm Tris-HCl; pH 8.0, 150 mM NaCl, 1 mM EDTA, 1.5 mM MgCl_2_, 10% glycerol, plus protease, and phosphatase inhibitors) and immunoprecipitated with anti-Syk antibody (N-19, Santa Cruz, Dallas TX). For Trem2 immunoprecipitation, cells were lysed with 1% n-Dodecyl-β-D-Maltoside and immunoprecipitated with anti-Trem2 (R&D systems, R&D Systems, Minneapolis, MN) or isotype control. Precipitated proteins were fractionated by SDS-PAGE, transferred to PVDF membranes, and probed with anti-phosphotyrosine antibody (4G10, Millipore, Burlington MA) and anti-Dap12 antibody (Cell Signaling Technology, Danvers MA). To confirm that all substrates were adequately immunoprecipitated, immunoblots were reprobed with anti-Syk antibody (Abcam, Cambridge, UK). Because the anti-Trem2 antibody does not detect Trem2 in immunoblotting, each cell lysate used for Trem2 immunoprecipitations contained equal amount of proteins with a control antibody (anti-actin, Santa Cruz, Dallas TX).

*Intracranial Administration*. Twenty-four 5-month-old male 5XFAD mice were assigned to one of two injection groups: MOPC (control antibody; mIgG1 isotype; BioXCell) or AL002a (anti-Trem2 antibody, mIgG1isotype; Alector). On the day of surgery, mice were weighed, anesthetized with isoflurane, and placed in a stereotaxic apparatus (51733D digital dual manipulator mouse stereotaxic frame; Stoelting, Wood Dale IL). A mid-sagittal incision was made to expose the cranium, and four burr holes were drilled with a dental drill mounted in the stereotaxic frame over the frontal cortex and hippocampus to the following coordinates: frontal cortex, anteroposterior, + 2.0 mm, lateral ±2.0 mm; hippocampus, anteroposterior − 2.7 mm; lateral, ± 2.5 mm, all taken from bregma. A 26-gauge needle attached to a 10-μl Hamilton syringe containing the solution to be injected was lowered 3.0 mm ventral to bregma, and a 2-μL injection was made over a 2-min period. Antibodies were diluted to a final concentration of 5 mg/ml in 1X PBS. The incision was cleaned and closed with surgical staples. Buprenorphine hydrochloride diluted to 0.015 mg/ml was intraperitoneally injected immediately post-surgery at 0.1 mg/kg dose per body weight. Animals received subsequent doses every 12 h until sacrifice. The tissue was harvested 72 h post-injection.

### Systemic administration

The two antibodies (MOPC; mIgG1 isotype; BioXCell, or AL002a; mIgG1isotype; Alector) were diluted to a final concentration of 5 mg/ml in 1XPBS. Twenty-four male 5XFAD (*N* = 12 per antibody group) and 15 wildtype mice (*N* = 8 in AL002a group and *N* = 7 in MOPC group) aged 4 months received AL002a, or MOPC control antibody at a dose of 50 mg/kg/week administered intraperitoneally for 14 weeks. Mice were tested in our behavioral paradigms during the 2 weeks prior to sacrifice. Mice were euthanized and tissue was harvested 24 h after the last injection.

### Tissue processing

Mice were perfused intracardially with 25 mL of normal saline. The brains were rapidly removed and bisected in the mid-sagittal plane. The left half was immersion fixed in freshly prepared 4% paraformaldehyde for 24 h. The right half was dissected into the cerebral cortex (anterior and posterior), hippocampus, striatum, and cerebellum. The dissected pieces of brain were flash frozen in liquid nitrogen and stored at − 80 °C. The left hemibrain was passed through a series of 10, 20, and 30% sucrose solutions for 24 h each as cryoprotection. Twenty-five micrometers of frozen horizontal sections was collected using a sliding microtome with a freezing stage and stored floating in PBS containing sodium azide at 4 °C. Sections were collected sequentially for the intracranial study and serially for the intraperitoneal study.

### Histology and immunohistochemistry

For the intracranial study, six sections spaced 1200 μm apart and spanning the estimated injection site were initially mounted and stained by mouse IgG to identify the injection site. For all subsequent histology and immunohistochemistry on the intracranial study, six sections spanning the injection site, each spaced approximately 100 μm apart, were selected and analyzed. For the systemic administration study, eight serial, horizontal sections spaced 1200 μm apart were selected for histology and immunohistochemistry. Sections were mounted onto slides and stained for Congo red as described previously [39]*.* Free-floating immunohistochemistry for CD11b (rat monoclonal; AbD Serotec), mouse IgG, and Aβ (rabbit polyclonal Aβ1–16; Invitrogen) was performed. Briefly, sections were quenched for endogenous peroxidase, blocked, and permeabilized. They were then incubated overnight in the primary antibody at 4 °C (Aβ 1:3000, CD11b 1:1000). After washing, sections were incubated for 2 h in the appropriate biotinylated secondary antibody (goat anti-rabbit IgG for Aβ 1:3000, goat anti-rat for CD11b 1:3000, goat anti-mouse IgG 1:3000; Vector Laboratories, Burlingame, CA, USA). For anti-mouse IgG, we skipped the overnight primary incubation and went straight from permeabilization to the 2-h secondary antibody incubation. Sections were then washed and incubated for 1 h in ABC. DAB with (CD11b) and without (Aβ) nickel were used for color development. Stained sections were mounted, air-dried overnight, dehydrated, and coverslipped in DPX (Electron Microscopy Sciences, Hatfield, PA, USA). Immunohistochemical analysis was performed by measuring the percent area occupied by positive stain using the Nikon Elements BR image analysis system (Melville, NY, USA) as described previously [40].

### Microglial clustering around plaques

We performed double labeling of eight serial, horizontal sections spaced 1200 μm apart to detect plaques and microglia using CD11b immunohistochemistry counter-stained with Congo red. Using a macro developed in our image analysis software, eight plaques restricted to a size range of 7–10-μm diameter were identified by the software in each of the frontal cortex and hippocampus. A ring was projected around the perimeter of the plaque that was 2 cell bodies wide (15 μm). The blinded analyst then clicked on each CD11b-positive cell body within the perimeter to determine the numbers of microglia surrounding that plaque. Between six and eight sections per mouse were analyzed in this way. The number of sections analyzed was variable because some small sections became folded and were not suitable for analysis. The mean number of microglia per plaque was calculated for each animal before being analyzed statistically as described below.

### Quantitative real-time reverse transcription (RT)-PCR

RNA was extracted from the left hippocampus using the EZNA RNA II Purification System (Omega Bio-Tek) according to the manufacturer’s instructions. RNA was quantified using the BioSpec Nano spectrophotometer (Shimadzu), and cDNA was reverse transcribed using the cDNA High Capacity kit (Applied Biosystems) according to the manufacturer’s instructions. Real-time RT-PCR was performed using the 384-well microfluidic card custom TaqMan assays containing TaqMan Gene Expression probes for our genes of interest (Applied Biosystems, Invitrogen). All gene expression data were normalized to 18S rRNA expression. Fold change was determined using the ^ΔΔ^Ct method.

### Beta-amyloid biochemistry

Protein was extracted for Aβ analysis from the right frontal cortex using a two-step extraction method. First, the brain was homogenized in PBS containing a complete protease and phosphatase inhibitor (Pierce Biotechnology Inc. Rockford, IL). These samples were centrifuged at 16,000×*g* at 4 °C for 1 h. The supernatant was removed and became the “soluble” extract. The resulting pellet was homogenized in 100 μl of 70% formic acid and centrifuged at 16,000×*g* at 4 °C for 1 h. The supernatant was removed and neutralized 1:20 with 1 M Tris-HCl and became the “insoluble” extract. Protein concentration for both the soluble and insoluble extracts was determined using the bicinchoninic acid (BCA) protein assay according to manufacturer’s instructions (Thermo Scientific, Rockford IL). We used the Meso-Scale Discovery multiplex ELISA system to measure Aβ38, Aβ40, and Aβ42 (MSD, Gaithersburg MD). MSD ELISA kits were run according to the manufacturer’s instructions.

### Radial arm water maze

After 12 weeks of treatment, mice were subject to a 2-day radial arm water maze (RAWM) paradigm, as previously described [41]. On day 1, groups of four mice performed 15 trials that were run in two sets of six trials followed by the last three trials. After each set, a second group of four mice was run, providing a rest period for the first group. Extra wait time was added to the end of the set of three trials to ensure the rest period was similar throughout the behavioral assessment. The goal arm was different for each mouse in a cohort to minimize odor cues, but the goal arm remained the same for a given mouse throughout the testing period. The start arm was varied for each trial. For the first 11 trials, the platform was alternately visible then hidden, and all subsequent trials used a hidden platform. The number of errors (incorrect arm entries) was measured in a 1-min time frame. As standard practice, mice failing to make an arm choice in 15 s were assigned one error. In order to minimize the influence of individual trial variability, each mouse's errors for three consecutive trials were averaged producing five data points (termed “blocks”) for each day, which were then analyzed statistically by ANOVA using the JMP statistical analysis program (SAS).

### Novel object recognition

On the third day, following the 2 days of RAWM, the novel object recognition task was performed. During the habituation phase, each mouse was gently placed into a square box (50 × 50 × 15 cm) for 30 min per day for 1 day without any objects. During the training phase, two identical objects, A1 and A2, were placed parallel to and near one wall of the square box. Each mouse was placed singly in the box and allowed to explore the objects for 5 min. Exploratory behavior was defined as directing the nose at the object at a distance of less than 2 cm and/or touching the object with the nose. The mouse was then returned to its home cage with a 1-h inter-trial interval. Both objects were replaced; one being a familiar object (A1) and the other a novel object (B). The mouse was returned to the box and allowed to explore the objects for 5 min during the test phase. Novel and familiar objects were alternated between the left and right positions to reduce potential bias toward a particular location. The objects and the box were cleaned with ethanol (10%) after each individual trial to eliminate olfactory cues. The exploration time (s) for each object in the trials was recorded. The preferential index (PI) was calculated as [time spent exploring novel object/total exploration time].

### Analysis

Data are presented as mean ± SEM. Statistical analysis was performed using the JMP statistical analysis program (SAS). Statistical significance was assigned where the *p* value was lower than 0.05. One-way ANOVA and two-way ANOVA were used, where appropriate, to detect treatment differences and differences within treatment groups along the time course.

## Results

### Trem2 signaling with AL002a

**Fig. 1:**
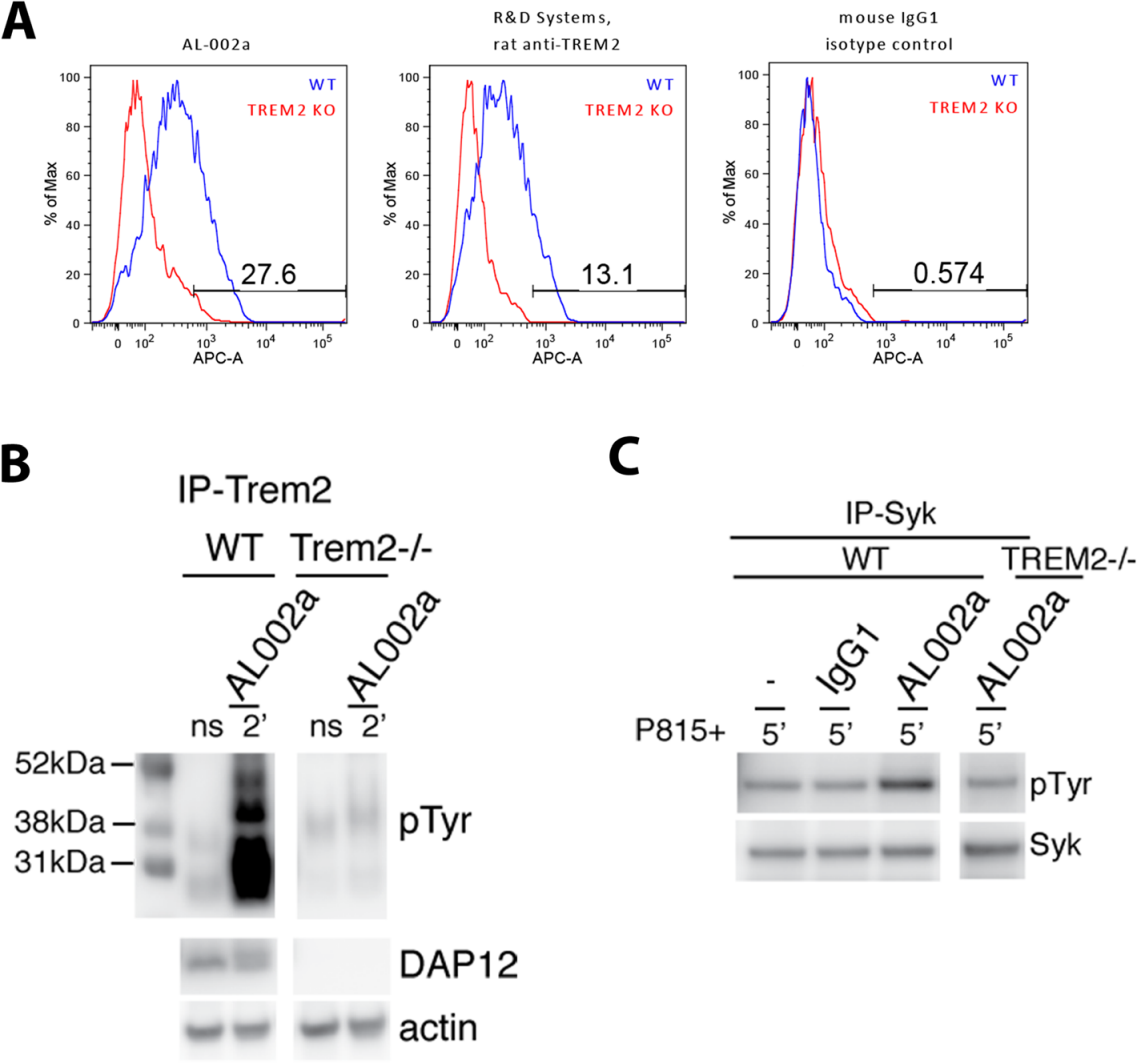
AL002a promoted TREM2-dependent DAP12 and Syk phosphorylation. Panel **a** shows that AL002a binds to BMM derived from WT, but not Trem2 KO mice, as measured by FACS. A rat anti-Trem2 antibody from R&D Systems was used as a positive binding control and an isotype msIgG1 antibody as a negative control. Panel **b**: Following AL002a stimulation, WT or Trem2-/- BMM were lysed and immunoprecipitated with Trem2 antibody. Protein was loaded on a SDS gel in unreduced conditions. The membranes were first blotted with anti-phosphotyrosine antibody and later stripped and blotted again with anti-Dap12 antibody and anti-actin. Panel **c**: After AL002a stimulation, WT or TREM2-/- BMM were lysed and immunoprecipitated with Syk antibody. The membranes were first blotted with anti-phosphotyrosine antibody and later stripped and blotted again with anti-Syk antibody

AL002a is a mouse IgG1 antibody that has been generated to recognize the extracellular portion of the TREM2 receptor. AL002a specifically recognizes Trem2 on WT bone marrow-derived macrophages (BMM), while the binding is reduced to isotype control levels in cells derived from Trem2 KO mice (Fig. 1a). In vitro, AL002a activates the Trem2 signaling pathway. Treatment of peripheral bone marrow-derived macrophages (BMM) with AL002a resulted in phosphorylation of both DAP12 and Syk, indicating activation of the Trem2 signaling pathway. Importantly, when these studies were repeated using BMM from Trem2-/- mice, there was no DAP12 or Syk response, indicating specific action through Trem2 (Fig. 1b, c).

### Intracranial administration of AL002a

**Fig. 2:**
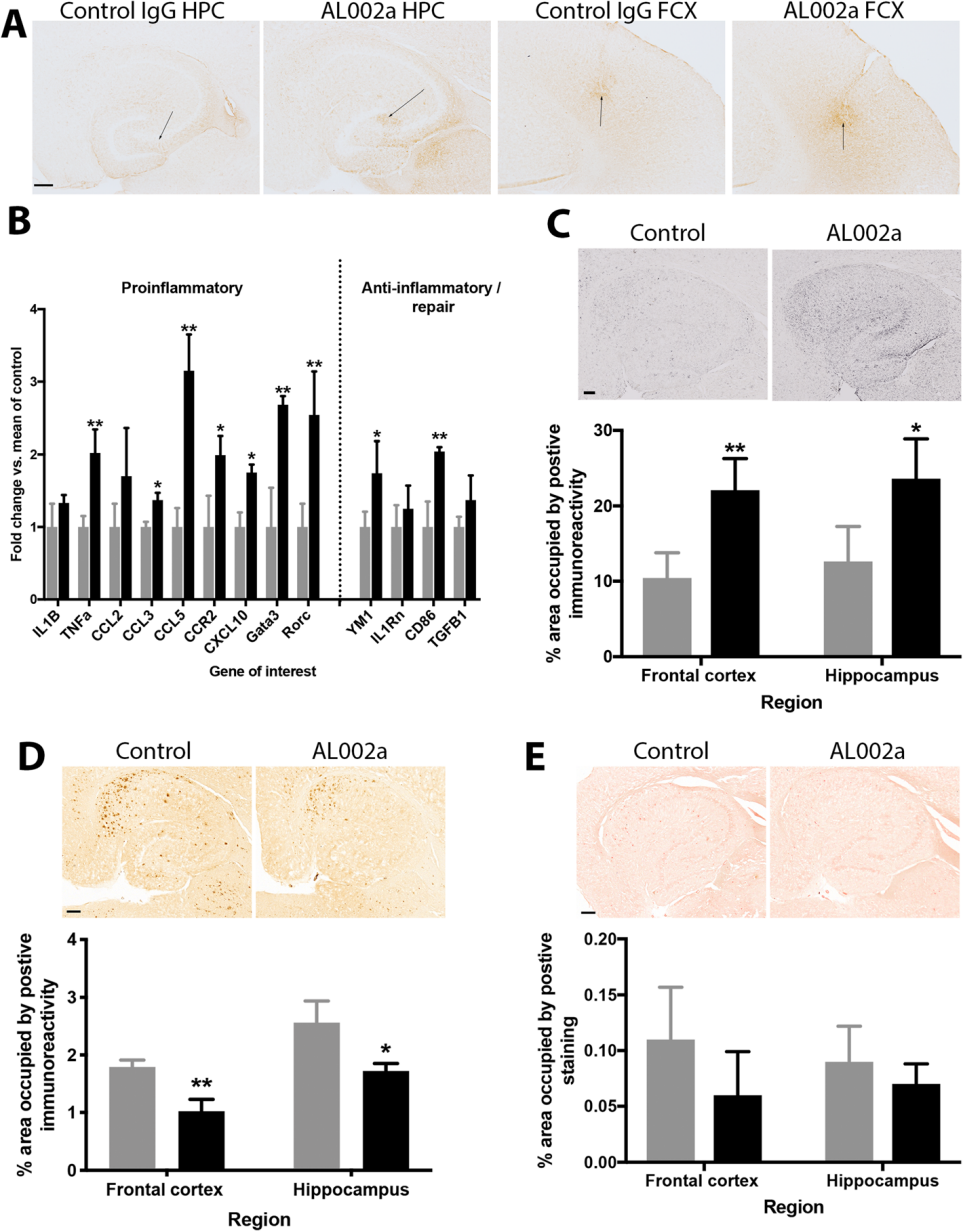
Intracranial injection of AL002a activates microglia and ameliorates amyloid deposition in 5XFAD mice. Panel **a** shows the anti-mouse IgG immunohistochemistry in the frontal cortex and hippocampus of mice receiving intracranial injection of either control IgG or AL002a. Arrows indicate the exact injection site. Panel **b** shows the RT-PCR data obtained from the right hippocampus of the injected mice. All data are shown as a fold-change relative to the mean of the control antibody-injected mice. Data were analyzed using single, one-way ANOVA measures for each gene of interest, using a Bonferroni correction for multiple comparisons. Panel **c** shows the microglial activation (CD11b) in the hippocampus (images shown) and frontal cortex following intracranial injection of control antibody or AL002a. Panel **d** shows the total Aβ deposition in the hippocampus (images shown) and frontal cortex following intracranial injection of control antibody or AL002a. Panel **e** shows Congo red labeling of compact amyloid deposits in the hippocampus (images shown) and frontal cortex following intracranial injection of control antibody or AL002a. For all graphs, black bars represent AL002a while gray bars represent control antibody. *P < 0.05, **P < 0.01. For images in **a**–**e**, magnification = ×40, scale bars shown = 120 μm

To determine the effects of AL002a in the brain, we performed stereotaxic surgery to inject 2 μl of 5 mg/ml AL002a or the isotype control antibody, MOPC, into the frontal cortex and hippocampus bilaterally (*N* = 12/antibody). In previous studies working with anti-Aβ antibodies, a time-course revealed the optimal time-point to examine the brain post-injection is 72 h [42]. Using that study as our guide, mice survived for 72 h and upon euthanasia, the right hippocampus was flash frozen and RNA was extracted to perform gene expression analysis. The left hemisphere was immersion fixed in paraformaldehyde and processed for histology. We performed immunohistochemistry on tissue sections to identify the location of the injection site and the spread of antibodies. As in our previous report with the intracranial injection of Aβ antibodies, we found the injected antibody did not spread far from the injection site (Fig. 2a); hence, we stayed within a 600-μm range of tissue sections for our analyses. Given we hypothesized that Trem2 activation would modulate the immune response in the brain, we used RNA extracted from the right hippocampus to perform real-time RT-PCR for inflammatory genes. The data are shown as a fold change from the 5XFAD mice receiving control IgG (Fig. 2b). We found at 72 h, there was a significant increase in both pro-inflammatory (IL1β, TNFα, CCL3, CCL5, CCR2, CXCL10, Gata3, Rorc) and anti-inflammatory (YM1, CD86) mediators as a result of AL002a treatment compared to mice injected with control antibody. To determine microglial activation, we performed immunohistochemistry for CD11b, which labels both activated and resting microglia. Activated microglia express greater levels of CD11b and cover a greater area due to the enlarged cell bodies and thickened processes associated with activation. We found a significant increase in CD11b immunoreactivity in 5XFAD and WT mice treated with a Trem2-agonizing antibody (AL002a) compared to the IgG1 isotype control (MOPC) treated mice, suggestive of microglial activation in the regions injected with AL002a compared to the same regions injected with control antibody (Fig. 2c).

The deposition of Aβ occurs as both diffuse and compact plaques with the vast majority of Aβ deposited being diffuse. Immunohistochemistry for total Aβ, which detects both compact and diffuse deposits, in 5XFAD transgenic mice receiving control antibody revealed a typical staining pattern for mice of this age (Fig. 2d) [38]. Conversely, compared to mice receiving the control antibody, mice receiving the AL002a antibody exhibited significant reductions in total Aβ immunohistochemistry in both the frontal cortex and hippocampus. In the frontal cortex, Aβ deposition was reduced by 40%, and in the hippocampus, Aβ deposition was reduced by 30% (Fig. 2d). The histological dye Congo red labels only compact amyloid deposits and stains approximately 10% of the material stained by immunohistochemistry for total Aβ at this age. As shown in Fig. 2e, the distribution of Congophilic deposits resembles that observed for total Aβ. Mice receiving the AL002a antibody showed non-significant reductions in Congo red labeling when compared to those mice receiving the control antibody (Fig. 2e).

### Systemic administration of AL002a

**Fig. 3:**
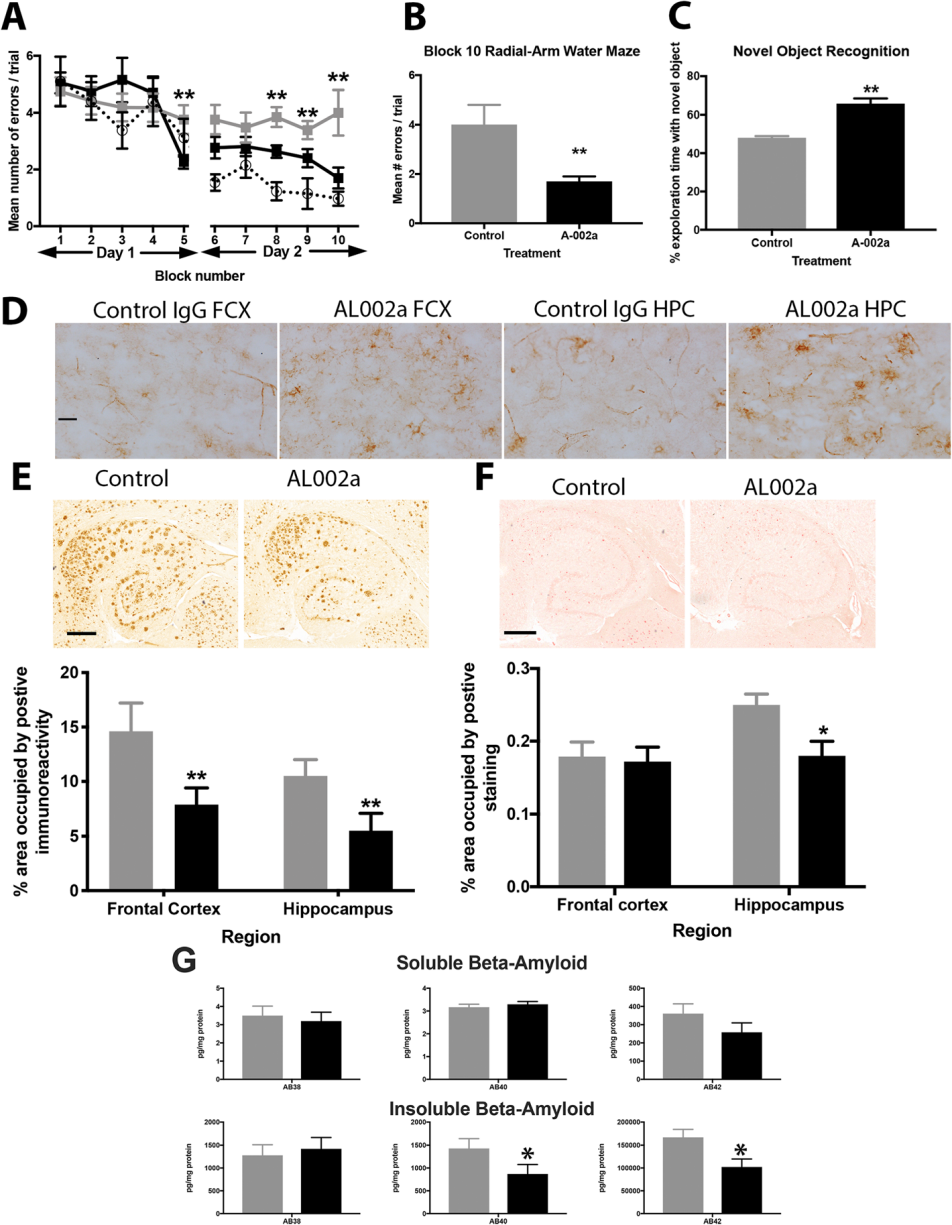
Systemic administration of AL002a improves learning and memory and lowers amyloid deposition in 5XFAD mice. Panel **a** shows the radial-arm water maze data. Blocks 1–5 are day 1, and blocks 6–10 are day 2. The black line is the learning curve of the 5XFAD mice receiving AL002a, the gray line is the learning curve of the 5XFAD mice receiving control antibody, and the dashed line is the learning curve of the non-transgenic littermates receiving control or AL002a (data was pooled due to lack of difference between the two treatment groups). Panel **b** shows a bar graph to illustrate the difference in number of errors made for the final block of trials (block 10). Panel **c** shows the bar graph for the novel object recognition data, where % exploration time with novel object is shown. Fifty percent of the exploration time would represent chance. Panel **d** shows anti-mouse IgG immunohistochemistry from the frontal cortex and hippocampus of mice receiving either control or AL002a antibodies, magnification = ×200, scale bar = 25 μm. Panel **e** shows the total Aβ deposition in the hippocampus (images shown) and frontal cortex of control antibody or AL002a. Panel **f** shows Congo red labeling of compact amyloid deposits in the hippocampus (images shown) and frontal cortex of control antibody or AL002a. Panel **g** shows the biochemical assessment of soluble and insoluble beta-amyloid 38, 40, and 42 in the frontal cortex as measured using the Meso-Scale Discoveries multiplex ELISA. For all graphs, black bars are AL002a, and gray bars are control antibody. *P < 0.05, ** *P* < 0.01. For images in **e** and **f**, magnification = ×40, scale bars shown = 120 μm

Given the positive outcomes of our stereotaxic studies above, we moved to perform a more clinically relevant, chronic, systemic administration study. Twenty-four male 5XFAD and fifteen male wildtype mice aged 4 months received AL002a (*N* = 12 5XFAD, *N* = 8 WT) or MOPC control antibody (*N* = 12 5XFAD, *N* = 7 WT) at a dose of 50 mg/kg/week administered intraperitoneally for 14 weeks. Mice were tested in our behavioral paradigms during the 2 weeks prior to sacrifice. The radial-arm water maze (RAWM) is a behavioral test that reliably detects spatial learning and memory deficits in aged transgenic mice [41]. 5XFAD transgenic mice were tested after 12 weeks of treatment with either AL002a or control antibody (MOPC). Included in the task were age-matched non-transgenic littermate mice treated with AL002a or control antibody (these mice were combined due to no observable difference between the two treatments in the non-transgenic mice). We found that the 5XFAD transgenic mice receiving control antibody were significantly impaired when compared with the non-transgenic mice (Fig. 3a). However, 5XFAD transgenic mice treated with AL002a performed significantly better than control-treated 5XFAD transgenic mice (Fig. 3a). The AL002a-treated 5XFAD mice were indistinguishable from the WT mice at the end of the second day of testing, averaging less than one error, our criterion for the stable acquisition of this task (Fig. 3a). In examining the AL002a effect at the end of the second day of testing, we found that there was a significant reduction in the number of errors in the 5XFAD mice receiving AL002a compared to 5XFAD receiving control antibody (Fig. 3b). One week after completion of the RAWM, the novel object recognition (NOR) task was performed to investigate recognition memory [43]. We found that 5XFAD transgenic mice treated with the control antibody spent significantly more time on the familiar object compared to the 5XFAD mice treated with AL002a (Fig. 3c).

To detect IgG in the brain, we performed immunohistochemistry for mouse IgG. We found that mice receiving AL002a showed IgG labeling of cells with a glial appearance, most likely microglia, which would be expected given the antibody’s specificity for Trem2 (Fig. 3d). Our control antibody-treated mice showed only vessel labeling and some non-specific labeling in the tissue, which could reflect non-specific labeling of the amyloid deposits in the tissue (Fig. 3d). Similar to what was observed in our stereotaxic study above, immunohistochemistry detecting total Aβ, which detects both compact and diffuse deposits, in the 5XFAD transgenic mice treated with control antibody showed a typical staining pattern for mice of this age (Fig. 3e) [38]. The 5XFAD mice treated with AL002a showed significant reductions in total Aβ immunohistochemistry in both the frontal cortex and hippocampus compared to control treatment (Fig. 3e). In the frontal cortex, Aβ deposition was reduced by 40%, and in the hippocampus, Aβ deposition was reduced by 35% (Fig. 3e). The distribution of Congophilic deposits resemble that observed for total Aβ (Fig. 3f); however, we found that as the 5XFAD accumulate amyloid deposition, Congophilic material did not significantly increase like a total amyloid deposition. The 5XFAD mice treated with AL002a showed significant reductions in compact amyloid deposits in only the hippocampus with no significant change in the frontal cortex (Fig. 3f). Assessment of beta-amyloid in the soluble and insoluble form isolated from the frontal cortex showed that insoluble Aβ40 and Aβ42 were significantly reduced by AL002a treatment, while soluble and insoluble Aβ38 remained unchanged, as did soluble Aβ40 and Aβ42.

**Fig. 4:**
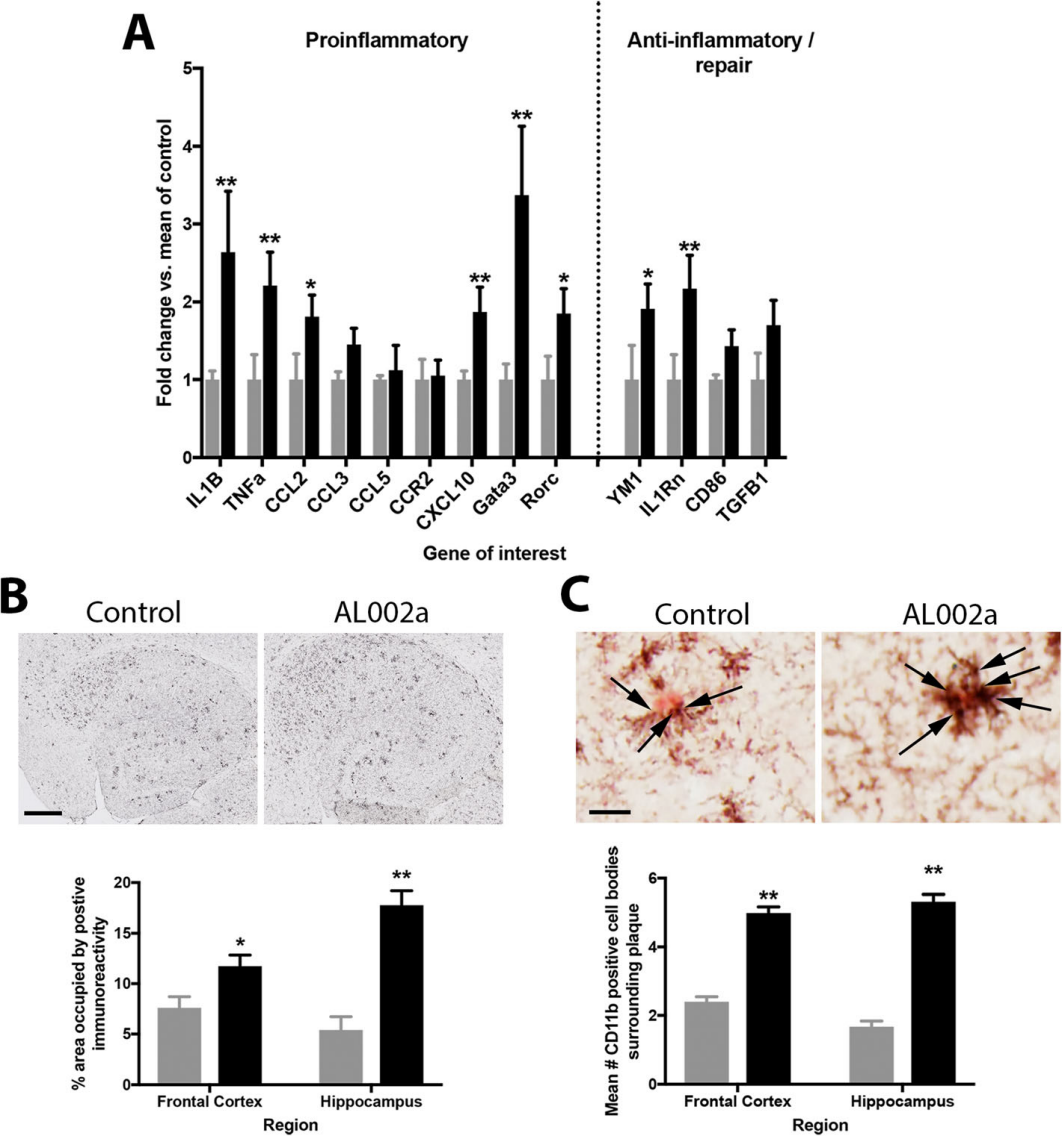
Panel **a** shows RT-PCR data obtained from the right hippocampus. All data are shown as fold-change relative to the mean of the control antibody treated mice. Data were analyzed using single, one-way ANOVA measures for each gene of interest, using a Bonferroni correction for multiple comparisons. Panel **b** shows the microglial activation (CD11b) in the hippocampus (images shown) and frontal cortex of control antibody or AL002a. Magnification = 40×, scale bar shown = 120 μm. Panel **c** shows the microglial clustering around an amyloid plaque. Arrows highlight microglial cell bodies. Magnification = 400×, scale bar shown = 12.5 μm. For all graphs, black bars are AL002a, and gray bars are control antibody. *P < 0.05, **P < 0.01

To characterize the neuroinflammatory response to AL002a, we isolated RNA from the right hippocampus and performed real-time RT-PCR for genes relatively specific for inflammatory and anti-inflammatory properties. The data in Fig. 4a are shown as a fold change from the 5XFAD mice treated with the control antibody. We found after 14 weeks of treatment, there was a significant increase in both pro-inflammatory (IL1β, TNFα, CCL2, CXCL10, Gata3, Rorc) and anti-inflammatory phenotypic markers (YM1 and IL1Rn) compared to control-treated mice (Fig. 4a). Immunohistochemistry detecting CD11b indicated a significant increase in CD11b positive staining in 5XFAD mice treated with AL002a compared to the 5XFAD mice treated with control antibody (Fig. 4b). During image processing, it was noted that there appeared to be more cells associated with plaques in some mice as compared to others (Fig. 4c). Using an analysis method developed for this purpose, we calculated the mean number of CD11b-positive cells per plaque for each animal. We found significantly increased numbers of CD11b-positive cells associated with plaques in mice treated with AL002a compared to mice treated with control antibody (Fig. 4c). The increase was approximately double the number of CD11b-positive cells in the frontal cortex and triple the number of microglia in the hippocampus per plaque.

## Discussion

Therapeutic approaches to the treatment of AD continue to focus on the major pathological hallmarks of the disease: amyloid plaques and neurofibrillary tangles [44–46]. These two pathologies remain the requirements for the diagnosis of AD. However, the explosion of genetic data has suggested that the risk for sporadic AD is driven by several distinct pathways such as neuroinflammation, membrane turnover and storage, and lipid metabolism [47]. Of particular importance was the description that a mutation in the TREM2 gene significantly increases an individual’s risk of developing AD [5, 6]. While this mutation has low penetrance in the population, those who carry the TREM2 R47H loss of function mutation have a 4.5-fold increased risk of developing AD compared to those without the mutation. Our hypothesis in the current study was that targeting TREM2 with an antibody, thereby activating the receptor, would increase TREM2 function leading to immune modulation, clearance of amyloid deposition, and improved cognition without the need to directly target the Aβ peptide itself. In testing this hypothesis, the antibody AL002a, developed by Alector, was found to activate Trem2 signaling in vitro and activate immune responses in vivo when injected intracranially or intraperitoneally. AL002a also activated microglial cells, increased clustering of microglia around the amyloid plaques, and ultimately resulted in reduced amyloid deposition and improved cognition (cognition was only examined in the systemic administration study). We cannot rule out systemic effects of the antibody treatment when injected intraperitoneally. While we tried to examine plasma for inflammatory mediators, we had insufficient sample to detect cytokines. Our future studies will prioritize this outcome measure. Importantly, during the performance of these studies and preparation of this manuscript, a publication by Schlepckow et al. demonstrated that the 4D9 monoclonal TREM2 antibody enhances protective microglial activities, reduces amyloid deposition, and stabilizes TREM2 on the cell surface [48].

It is not unusual to find an association between the microglial activation and amyloid reductions. As far back as 2000, lipopolysaccharide (LPS), the prototypical immune activator, was found to activate microglia and reduce amyloid deposition [49]. Anti-Aβ antibodies also activate microglia and reduce amyloid deposition [42, 50]. It is interesting to compare and contrast the findings of AL002a to an anti-Aβ antibody treatment approach currently in clinical trials. We have previously shown that anti-Aβ immunotherapy activates microglia [50–52] and alters neuroinflammatory gene expression [53], but we find a unique inflammatory signature with AL002a as opposed to the anti-Aβ antibody. AL002a increased both pro-inflammatory and anti-inflammatory/repair gene expression, while anti-Aβ antibodies have only been shown to increase pro-inflammatory gene expression and, in some cases, decrease the anti-inflammatory gene expression. It is possible that the increase in gene expression by AL002a represents a more homeostatic neuroinflammatory response with a more limited capacity to induce the surrounding tissue damage while also ameliorating the amyloid deposition. This is purely speculative, however, given the limited number of genes examined in the current study. A more unbiased assessment of gene expression in the future could address this issue more fully. We do not believe that our observation is a non-specific IgG response considering we are showing gene expression differences between AL002a and control antibody, which were injected at the same concentration. We also explored whether the antibody is modulating sTrem2 levels, but could not detect circulating Trem2 in the plasma. Future studies will collect more plasma to allow us to perform detailed analyses of the systemic response to this treatment.

We found immune-associated genes being expressed in different ways depending on the route of administration. Following the intracranial administration of AL002a, we found almost every gene measured was increased compared to the age-matched 5XFAD mice receiving an intracranial injection of control antibody. This likely reflects the acute response to the antibody and activation of Trem2. In contrast, following 14 weeks of systemic administration, the chemokines CCL3, CCL5, and CCR2 were not significantly increased whereas IL1β and IL1Rn were significantly increased. CCR2 is a chemokine receptor expressed on microglia thought to mediate the accumulation of phagocytes at sites of inflammation [54]. CCL2 and CCL5 are chemokines that have been shown to increase the chemotaxis of microglia toward amyloid deposits [55]. The increased CCL2 and CCL5 expression, along with the increased CCR2 expression could be responsible, in part at least, to the increased clustering of microglia around the amyloid deposits seen in our systemic administration study. We also found increases in two key genes that are associated with T cell differentiation into Th17 cells; Gata3 and Rorc [56]. While increased expression of IL1β and TNFα are sometimes associated with tissue damage and neurodegeneration, in some settings, the increase in these pro-inflammatory cytokines accompanies the clearance of pathological proteins such as amyloid deposits [57, 58]. Anti-Aβ antibodies have been shown to increase these cytokines [53], as has genetic deletion of IL-10 [59] and injection of LPS [49, 60]. Additionally, the reductions in these cytokines have been associated with worse cognitive outcomes and exacerbation of amyloid deposition as observed when IL10 is over-expressed [61], or when lithium is administered to mice, which enhances IL-10 signaling [62]. It is our hypothesis that the enhanced expression of inflammatory genes in the current setting is balanced by the YM1, IL1Rn, and CD86 expression, thereby limiting the capacity for tissue damage. Future studies will further examine this hypothesis. It is also likely that some of the differences between our intracranial and systemic administration studies reflects the difference between an acute inflammatory response including a stab wound from the needle and a slower response to smaller levels of the antibody in the brain, accumulating over time.

By using two distinct behavioral paradigms in the current study, we are confident in concluding that AL002a significantly enhances cognition, or at the very least, prevents progression of cognitive decline, in the 5XFAD mouse model. The 2-day radial arm water maze task was designed to test both working memory (the day 1 learning) and long-term spatial memory (day 1 to day 2 retention). The novel object recognition task, as used in the current study, is a useful task to assess short-term memory. Both tasks have been shown to be heavily hippocampal dependent, but also have aspects of cortical involvement [63, 64]. In contrast to the radial arm water maze, the novel object recognition does not rely on motivation or reward, but simply on the innate exploratory behavior of a mouse. We found robust improvements in cognition with AL002a systemic administration as detected in either the radial arm water maze task or the novel object recognition task.

## Conclusion

In summary, here we show that the therapeutic targeting of Trem2 using a Trem2-activating antibody leads to the activation of microglia, recruitment of microglia to amyloid plaques, reduced amyloid deposition, and ultimately, improved cognition. Trem2-deficient microglia *fail to fully activate into phagocytic*, disease-associated microglia and to express the associated gene signature *in amyloid-depositing mice.* Likewise, *Trem2-deficient microglia* fail to clear myelin debris. Our data support a critical role for Trem2 in microglial phagocytosis with increased microglial clustering at amyloid plaques and reductions in amyloid deposition using a Trem2-activating approach. We predict that activation of TREM2 through the use of antibodies like AL002a will prove to be a novel, innovative therapeutic approach to the treatment of AD that will lack the adverse events observed with direct binding of Aβ in the brain by anti-Aβ antibodies.

## Data Availability

All raw data is available upon request from the corresponding author. To obtain the TREM2 antibody, correspondence should be addressed to Alector.

## Abbreviations

Aβ: Amyloid-beta
AD: Alzheimer’s disease
BMM: Bone marrow-derived macrophage
CCL2: C-C motif chemokine ligand 2
CCL3: C-C motif chemokine ligand 3
CCL5: C-C motif chemokine ligand 5
CCR2: C-C motif chemokine receptor 2
CXCL10: C-X-C motif chemokine ligand 10
ERK: Extracellular signal-regulated kinases
FTD: Frontotemporal dementia
Gata3: GATA-binding protein 3
IL1β: Interleukin 1 beta
IL1RN: Interleukin 1 receptor antagonist
ITAM: Immunoreceptor tyrosine-based activation motif
LPS: Lipopolysaccharide
NFAT: Nuclear factor of activated T cells
NHD: Nasu-Hakola disease
NOR: Novel object recognition
PI3K: Phosphoinositide 3 kinase
PLOSL: Polycystic lipomembranous osteodysplasia with sclerosing leukoencephalopathy
RAWM: Radial arm water maze
Rorc: Nuclear receptor ROR-gamma
TGFβ1: Transforming growth factor 1 beta
TNFα: Tumor necrosis factor-alpha
TREM2: Triggering receptor expressed on myeloid cell-2
YM1: Chitinase-like protein 3
